# Immunoglobulin G (IgG) to IgM ratio in secondary adult dengue infection using samples from early days of symptoms onset

**DOI:** 10.1186/s12879-015-1022-9

**Published:** 2015-07-21

**Authors:** Cucunawangsih, Nata Pratama Hardjo Lugito, Andree Kurniawan

**Affiliations:** Microbiology Department, Faculty of Medicine, Pelita Harapan University, Jendral Sudirman Boulevard, Lippo Karawaci, Tangerang, Banten 15811 Indonesia; Internal Medicine Department, Faculty of Medicine, Pelita Harapan University, Jendral Sudirman Boulevard, Lippo Karawaci, Tangerang, Banten 15811 Indonesia

**Keywords:** Dengue, Secondary infection, IgG, IgM, ratio

## Abstract

**Background:**

Dengue virus (DENV) infection is an emerging arboviral infection in tropical and sub-tropical countries in South-East Asia, the Western Pacific and South and Central America. Secondary DENV infection is the most widely accepted risk factor for the development of severe forms. Methods to discriminate primary and secondary DENV infection may be of great prognostic value. ELISA based detection of specific antibodies (IgG and IgM) to the four DENV serotypes is valuable for detemination of primary or secondary infection. Several studies had been performed to discriminate primary and secondary DENV infection using the ratio of IgG and IgM at the various days of symptoms onset. The aim of this study is to determine the best cut-off point of IgG to IgM ratio is able to discriminating secondary from primary DENV infection in adult using samples from early days of symptoms onset.

**Methods:**

This prospective cohort study on 48 adult patients with DENV infected patients on the period of August 2011–January 2012 in 5 out-patient settings health facilities in Tangerang district, Banten province, Indonesia with chief complaint of fever less than 3 days. Datas were collected on the day the patients attended health facilities, consisted of demographic, clinical, laboratory, and serological data. Serological data from 48 serum sample from adult patients were evaluated using Focus Diagnostics Dengue Virus IgM and IgG Capture DxSelect™ ELISA Kits to determine IgG, IgM index values and SD Bioline Dengue Duo™ Rapid Tests to determine NS1, IgG, and IgM result.

**Results:**

According to NS1, IgG and IgM results, 36 patients were classified as secondary infection, 12 were primary Infection. The mean (SD) of IgG/IgM ratios for secondary and primary infection were 3.28 (0.54) and 0.18 (0.11) consecutively. The IgG/IgM ratio of ≥ 1.14 confirmed secondary infection with sensitivity of 80.56 %, specificity 91.67 %, accuracy level 83.33 %, and likely hood ratio of (LR) 9.67.

**Conclusion:**

The IgG/IgM ratio of ≥ 1.14 as the best cut off point to determine secondary DENV infection in early days of symptoms onset.

## Background

Dengue virus (DENV) infection is an emerging arboviral infection in tropical and sub-tropical countries in South-East Asia, the Western Pacific and South and Central America [1]. Up to 2.5 billion people globally live under the threat of DENV infection and its severe forms. Severe forms of DENV infection such as dengue hemorrhagic fever (DHF) and dengue shock syndrome(DSS) are mainly associated with secondary infection [2, 3]. Epidemiological studies found an association between more severe forms and secondary heterotypic infection with a distinct serotype from the primary infection [4, 5]. The lack of methods to predict which DENV infected patient will progress to its severe forms resulted in over-admission and over-treatment in hyperendemic area. Methods to discriminate primary and secondary DENV infection may be of great prognostic value in these area [6].

Although clinical evaluation is important for DENV infection diagnosis and treatment, enzyme-linked immunosorbent assays (ELISA) based detection of specific antibodies (immunoglobulin G (IgG) and IgM) to the four DENV serotypes is valuable for the diagnosis of acute infection and for detemination of primary or secondary infection [7, 8]. The IgG and IgM ELISA has the advantage of being easier to perform, as well as being suitable for surveillance and large-scale studies. Many commercial and standardized ELISA tests for both IgM and IgG antibody detection have also become available. Several studies had been performed to discriminate primary and secondary DENV infection using the ratio of IgG and IgM at the various days of symptoms onset [9–13]. Different ratios found in these studies might be due to different settings and seroepidemiologics [9, 10]. The aim of this study was to determine the best cut off point of IgG to IgM ratio for differentiating secondary from primary DENV infection in adult using samples from early days of symptoms onset.

## Methods

### Ethics statement

The Research and Research Ethical Expert Committee Faculty of Community Health, Universityof Indonesia granted ethics approval of the study (032/H2.F10/PPM.00/2011).

### Study population and sample

This prospective cohort study on 84 adult patients with DENV infected patients on the period of August 2011–January 2012 in five out-patient settings health facilities (two primary health care, two out-patient clinics and one hospital) in Tangerang district, Banten province, Indonesia with chief complaint of fever less than 3 days. All study participants were written informed consent and enrolled in the study. Datas were collected on the day the patients attended health facilities, consisted of demographic, clinical, laboratory, and serological data. Serological data from serum sample were evaluated using Focus Diagnostics Dengue Virus IgM and IgG Capture DxSelect™ ELISA Kits to determine IgG, IgM index values and SD Bioline Dengue Duo™ Rapid Tests to determine NS1, IgG, and IgM result.

### DENV IgG measurement

In the IgG ELISA assay, the polystyrene microwells are coated with equal proportions of inactivated and purified DENV types 1–4. Diluted serum samples and controls are incubated in the wells to allow specific antibody present in the samples to react with the antigen. Control, calibrator, and patient sera were diluted 1:101 in sample buffer, and 0.1 ml of diluted specimen was added to assigned microtiter wells. Nonspecificreactants are removed by washing and peroxidase-conjugated anti-human IgG isadded and reacts with specific IgG. Excess conjugate is removed by washing. Enzyme substrate and chromogen are added, and the color is allowed to develop. After adding the Stop Reagent, the resultant color change is quantified by a spectrophotometric reading of optical density (OD) which is directly proportional to the amount of antigen-specific IgG present in the sample. Absorbance was measured at 450 nm using an ELISA reader. Sample opticaldensity readings are compared with reference cut-off OD readings to determine results. An index value of > 1.00 is presumptive for the presence of IgG antibodies to DENV [14].

### DENV IgM measurement

In the IgM ELISA assay, the polystyrene microwells are coated with anti-human antibody specific for IgM (μ-chain). Diluted serum samples and controls are incubated in the wells, and IgM present in the sample binds to the anti-human antibody (IgM specific) in the wells. Control, calibrator, and patient sera were diluted 1:101 in sample buffer, and 0.1 ml of diluted specimen was added to assigned microtiter wells. Nonspecific reactants are removed by washing. DENV antigen is then added to the wells and incubated; and, if anti-DENV IgM is present in the sample, the antigen binds to the anti-DENV in the well. Unbound DENV antigen is then removed by washing the well. Mouse anti-DENV conjugated with horseradish peroxidase (HRPO) is then added to the wells and incubated; and, if DENV antigen has been retained in the well by the anti-DENV in the sample, the mouse anti-DENV: HRPO binds to the DENV antigen in the wells. Excess conjugate is removed by washing. Enzyme substrate and chromogen are added, and the color is allowed to develop. After adding the Stop Reagent, the resultant color change is quantified by a spectrophotometric reading of OD which is directly proportional to the amount of antigen-specific IgM present in the sample. Absorbance was measured at 450 nm using an ELISA reader. Sample optical density readings are compared with reference cut-off OD readings to determine results. An index value of > 1.00 is presumptive for the presence of IgM antibodies to DENV [15].

### DENV IgM/IgG ratio

The DV IgG/IgM ratio was calculated for sera positive for DV IgG and IgM by dividing the IgG index value with the IgM index value.

### Definition of acute primary and secondary DENV infections

Definition of primary and secondary DENV infection was based on serological patterns of a given patient. Primary infection was defined as a negative IgM and negative IgG or positive IgM and negative IgG. Secondary infection was defined as a negative IgM and positive IgG. Focus Diagnostics Dengue Virus IgM and IgG Capture DxSelect™ ELISA Kits were used to determine IgG and IgM results.

### Statistical analysis

Chi-square tests were used for categorical variables; independent *t*-test and Mann–Whitney U tests for continuous variables. Sensitivity, specificity, accuracy, and cut-off point for IgG/IgM ratio were determined using Receiver Operator Characteristic (ROC) curve. Statistical analyses were performed using SPSS Statistics 19th version (SPSS Inc, 2010).

## Results

A total of 84 adult patients with clinically sign of dengue illness were initially enrolled in the study. DENV infected patients were patients with positive results for NS1, IgG, or IgM; therefore only 48 serum samples were eligible for serological testing (Table 1). There are 36 patients were classified into secondary infection and 12 were primary infections by NS1 rapid test, IgG and IgM. According to NS1, IgG and IgM results, 36 patients were classified as secondary infection, 12 were primary Infection. Demographic, clinical, laboratory and serological data of patients with secondary and primary DENV infection are shown in Table 2.

**Table 1:** Data of patients with DENV infection (N = 48)

No.	Age	Sex	BMI	Platelet	NS1	IgM1 Index	IgM1	IgM2 Index	IgM2	IgG Index	IgG	Dengue Infection	IgG / IgM ratio	DEN1	DEN2	DEN3	DEN4
1.	34	M	33.05	85,000	P	0.553	N	2.647	P	2.675	P	2nd	4.84	N	N	N	N
2.	27	F	20.20	125,000	P	0.674	N	0.805	N	4.344	P	2nd	6.45	N	N	N	N
3.	41	M	17.30	118,000	P	0.208	N	0.038	N	3.999	P	2nd	19.23	N	P	N	N
4.	43	M	24.91	30,000	P	2.659	P	NA		4.886	P	2nd	1.84	N	P	P	N
5.	37	F	19.65	81,000	P	2.4	P	4.159	P	4.715	P	2nd	1.96	N	N	N	N
6.	19	F	17.31	127,000	P	0.724	N	NA		3.036	P	2nd	4.19	N	N	N	N
7.	24	M	20.43	129,000	P	0.499	N	1.359	P	0.1	N	1st	0.20	P	N	N	N
8.	45	F	21.21	130,000	P	7.52	P	NA		0.812	N	1st	0.11	P	P	N	N
9.	40	F	27.47	42,000	P	0.773	N	0.86	N	2.055	P	2nd	2.66	NA			
10.	20	M	22.22	37,000	P	4.702	P	NA		5.338	P	2nd	1.14	N	N	N	N
11.	24	M	21.09	254,000	N	1.08	P	NA		0.024	N	1st	0.02	N	N	N	P
12.	30	M	22.75	167,000	P	0.559	N	3.068	P	0.09	N	1st	0.16	P	P	N	N
13.	21	F	30.04	71,000	P	1.036	P	5.507	P	0.144	N	1st	0.14	N	N	N	N
14.	43	M	27.21	140,600	N	1.728	P	NA		4.321	P	2nd	2.50	N	P	N	N
15.	25	F	20.07	78,000	N	1.193	P	NA		4.072	P	2nd	3.41	P	N	N	N
16.	24	M	21.51	137,000	P	1.425	P	7.748	P	3.79	P	2nd	2.66	P	N	N	N
17.	33	F	23.00	128,200	P	1.167	P	NA		4.787	P	2nd	4.10	N	N	N	N
18.	32	F	21.30	97,000	P	4.981	P	6.329	P	4.586	P	2nd	0.92	N	N	N	N
19.	18	F	20.28	83,000	N	1.989	P	2.132	P	1.28	P	2nd	0.64	P	N	N	N
20.	43	M	23.87	112,000	P	1.742	P	9.167	P	3.018	P	2nd	1.73	NA			
21.	21	F	19.37	95,000	P	1.554	P	NA		5.119	P	2nd	3.29	N	N	N	N
22.	54	M	32.71	79,000	N	9.447	P	10.16	P	5.28	P	2nd	0.56	N	N	N	N
23.	17	F	16.65	148,000	P	0.507	N	NA		0.032	N	1st	0.06	P	N	N	N
24.	45	M	27.35	131,000	N	1.62	P	NA		3.602	P	2nd	2.22	N	N	P	N
25.	49	M	24.97	61,000	P	9.063	P	10.61	P	4.86	P	2nd	0.54	NA			
26.	20	F	29.70	215,100	P	2.762	P	5.321	P	4.789	P	2nd	1.73	P	N	N	N
27.	29	M	24.22	37,000	P	0.993	N	NA		4.372	P	2nd	4.40	N	P	N	N
28.	23	M	18.61	80,000	P	1.989	P	7.984	P	0.022	N	1st	0.01	N	N	N	N
29.	53	F	20.88	139,000	N	1.093	P	NA		2.792	P	2nd	2.55	N	P	N	N
30.	23	M	21.62	191,000	N	1.025	P	0.937	N	3.463	P	2nd	3.38	N	N	P	N
31.	25	F	22.63	108,000	P	1.304	P	3.238	P	0.06	N	1st	0.05	N	N	N	N
32.	40	M	26.98	224,400	P	1.189	P	5.967	P	3.534	P	2nd	2.97	P	N	P	N
33.	40	F	19.53	118,000	P	6.247	P	8.427	P	5.008	P	2nd	0.80	N	N	N	N
34.	30	M	20.90	114,000	P	0.663	N	1.37	P	1.724	P	2nd	2.60	P	N	N	N
35.	30	M	23.03	110,000	P	2.438	P	3.901	P	4.893	P	2nd	2.01	N	N	N	N
36.	16	F	21.45	87,000	P	3.222	P	6.033	P	3.689	P	2nd	1.14	N	P	P	N
37.	29	M	25.56	185,000	P	8.986	P	11.95	P	0.042	N	1st	0.00	N	N	N	N
38.	49	F	24.00	32,300	N	1.763	P	NA		4.634	P	2nd	2.63	N	P	P	N
39.	30	M	27.54	147,000	N	1.654	P	1.602	P	4.418	P	2nd	2.67	P	N	N	N
40.	18	M	20.76	63,000	N	5.95	P	NA		5.125	P	2nd	0.86	N	N	P	N
41.	31	F	19.97	38,000	N	1.615	P	1.833	P	4.863	P	2nd	3.01	N	N	N	N
42.	23	F	23.24	232,900	P	0.367	N	1.098	P	0.499	N	1st	1.36	P	N	N	N
43.	32	F	18.81	158,000	P	0.633	N	2.054	P	3.221	P	2nd	5.09	N	N	N	N
44.	22	M	24.56	158,400	P	2.498	P	7.228	P	0.027	N	1st	0.01	N	N	N	N
45.	20	F	18.35	133,000	P	7.528	P	NA		0.146	N	1st	0.02	N	P	N	N
46.	26	F	21.61	192,200	P	0.58	N	1.985	P	4.499	P	2nd	7.76	P	N	N	N
47.	18	M	15.40	151,000	P	0.793	N	2.615	P	4.52	P	2nd	5.70	N	N	N	N
48.	32	M	27.91	154,000	N	1.159	P	1.35	P	4.619	P	2nd	3.99	N	N	N	N

N-negative, P-positive, 1st-primary, 2nd-secondary

**Table 2:** Characteristics of patients with secondary (N = 36) and primary DENV infection (N = 12)

Demographic, clinical, and laboratory datas	Secondary infection	Primary infection
Sex, n(%)^a^		
Male	19 (52.8 %)	6 (50.0 %)
Female	17 (47.2 %)	6 (50.0 %)
Age (year), median (range)^b^	31.5 (16–54)	23.5 (17–45)
Body mass index (kg/m^2^), mean (SD)^c^	21.62 (15.40-33.06)	21.93 (16.65-30.04)
IgM index value, mean (SD)^c^	2.28 (0.37)	2.82 (0.93)
IgG index value, mean (SD)^c^	4.05 (0.17)	0.17 (0.07)
IgG/ IgM ratio, median (range)^c^	3.28 (0.54)	0.18 (0.11)
Platelet (/μl),mean (SD)^c^	109,938.9 (51,073.61)	149,691.7 (55,029.6)

^a^Chi-square test, ^b^Mann–Whitney *U* test, ^c^Independent *t*-test

**Fig. 1:**
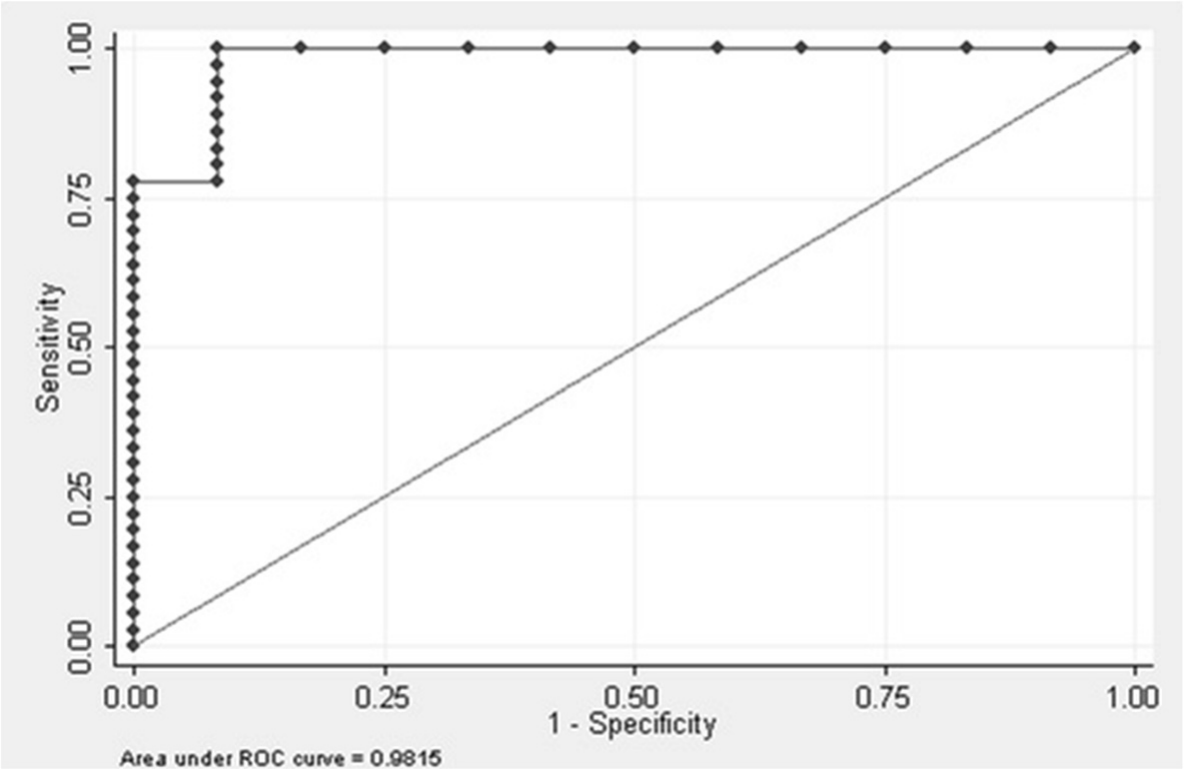
Receiver operator curve for IgG/IgM ratio

The study found that IgG/IgM ratio of ≥1.14 confirmed secondary infection with sensitivity of 80.56 %, specificity 91.67 %, accuracy level 83.33 %, and likelihood ratio of (LR) 9.67 as shown in Table 3. The cut-off point had good performance because area under ROC curve was 0.98 (Fig. 1).

**Table 3:** Diagnostic value of various cut-off point of the IgG/IgM ratio in determining secondary DENV infection

IgG / IgM ratio	Sensitivity	Specificity	Accuracy	LR+
≥0.92	86.11	91.67	87.50	10.33
≥1.13	83.33	91.67	85.42	10.00
≥1.14^a^	80.56	91.67	83.33	9.67
≥1.36	77.78	91.67	81.25	9.33
≥1.73	77.78	100.00	83.33	

^a^Best cut-off point

## Discussion

A primary DENV infection is defined as the absence of specific anti-dengue IgG antibodies in the first serum samples during the acute phase, with anti-dengue IgM, virus isolation and/or virus RNA being present, and dengue virus IgG being detected in a later sample. A secondary DENV infection is defined by the presence of specific anti-dengue IgG and the absence of anti-dengue IgM in the first sample, together with a positive RT-PCR and/or virus isolation, followed by the presence of anti-dengue IgM in a later sample [7].

Secondary DENV infection is the most widely accepted risk factor for the development of severe forms. But, there is still no reliable test to discriminate primary and secondary infections in the early days of disease. Differentiation of primary and secondary infections is essential, particularly in low resources endemic areas where re-infections with different serotypes are frequent. Immunoglobulin M and IgG dengue ELISA kits are commercially available at relatively low cost, so DENV infection diagnosis is now being done in many laboratories worldwide [16].

In primary infection both IgM and IgG test often give negative results in the first 5 days of symptoms onset [17]. In secondary infection, IgM was first detected on day 4, while IgG was detected in most of the samples on day seven or later, and in some secondary DENV infections, IgM is often not detected at all [18–21]. After day five in primary infection and day 6 in secondary infection, IgM and IgG were positive in most of the samples [20]. IgM-positive rate in secondary infection are not as high as in primary infection, whereas the IgG-positive rate is higher in secondary than in primary infection especially in early days of symptoms onset [18–21]. Thus, the ideal combination of tests to detect an acute DENV infection are the reverse transcriptase polymerase chain reaction (RT-PCR) and IgG test for the first 5 days after symptoms onset, and the IgM and IgG tests from the day 6 of symptoms and after. Defining primary and secondary infections according to these criteria is very expensive, and most clinical laboratories in dengue endemic area cannot perform all these tests [16].

A study using the PanBio kit found an IgG result of 40 PanBio units as the cut-off used to discriminate primary and secondary DENV infection [20]. Other study found an avidity IgG to discriminate primary and secondary DENV infection using a single acute-phase serum sample [22]. Other study used classification method to discriminate between primary and secondary dengue infections based on IgG antibody levels and the number of days of symptoms [18]. But results from that study stated that to correctly dis1criminate primary and secondary infection, combination of several tests, such as IgM and IgG levels, virus isolation and/or viral RNA detection is a necessity.

Several studies were done to discriminate primary and secondary DENV infection using the ratio of IgG and IgM at the various days of disease [9–13]. A study in Bali, Indonesia found IgG/IgM ratio of ≥ 1.1 on the day 5–7 of disease is a good predictor of secondary dengue infection. This study was done on children hospitalized for suspected DENV infection, with mean age of 6 year-old [23]. Study in Thailand using HI test as the gold standard found that the best cut off point of IgG/IgM ratio for secondary infection was ≥ 1.78 [9]. Study in Malaysia found that the best cut off point for secondary infection was ≥ 2.0[16]. This study found that IgG/IgM ratio of ≥1.14 as the best cut off point to determine secondary DENV infection, with sensitivity of 80.56 % and spesificity of 91.67 %. Different ratios found in these studies might be due to different settings and seroepidemiologics [9, 10, 20, 23].

Studies using samples from DENV infection patients in the Americas to identify acute primary and secondary infections did not use IgM and IgG titer-negative sera to determine the IgM/IgG OD ratio. A study using samples from IgM and IgG titer-negative sera found a cut off IgM/IgG OD ratio that was comparable with studies previously [9, 24]. This study also used samples from IgM and IgG titer-negative sera and also found a cut off IgG/IgM ratio comparable to a study previously [23].

## Conclusions

To discriminate primary and secondary DENV infection, combination of several tests such as IgM and IgG levels is a necessity. Immunoglobulin M and IgG dengue ELISA kits are commercially available at relatively low cost, so DENV infection diagnosis can be done readily particularly in low resources endemic areas where re-infections with different serotypes are frequent. This study found that IgG/IgM ratio of ≥1.14 as the best cut off point to determine secondary DENV infection in early days of symptoms onset.
